# P-98. Orthopedic Hand Infections: A 10 Year Review of Cases from Southern Arizona

**DOI:** 10.1093/ofid/ofaf695.327

**Published:** 2026-01-11

**Authors:** Timothy M Marshall, Jacob Denton, Esha V Rajadhyaksha, Maryam Emami Neyestanak, Pantea Sazegar, Ryan Garcia, Tolga Turker, Talha Riaz

**Affiliations:** University of Arizona College of Medicine Tucson, Tucson, Arizona; University of Arizona, Tucson, Arizona; University of Arizona College of Medicine - Tucson, Tucson, Arizona; University of Arizona, Tucson, Arizona; University of Arizona college of medicine, Tucson, Arizona; University of Arizona, Tucson, Arizona; The University of Arizona, Tucson, Arizona; University of Arizona College of Medicine Tucson, Tucson, Arizona

## Abstract

**Background:**

Orthopedic hand infections are a common complaint for patients seen by orthopedic surgeons and infectious diseases physicians. These can present as deep hand space infections, tenosynovitis, abscess or osteomyelitis, and as such treatment courses vary widely. The aim of our study was to characterize orthopedic hand infections in a large cohort of patients from Southern Arizona, assessing the risk factors, treatment and outcomes.

**Figure:**
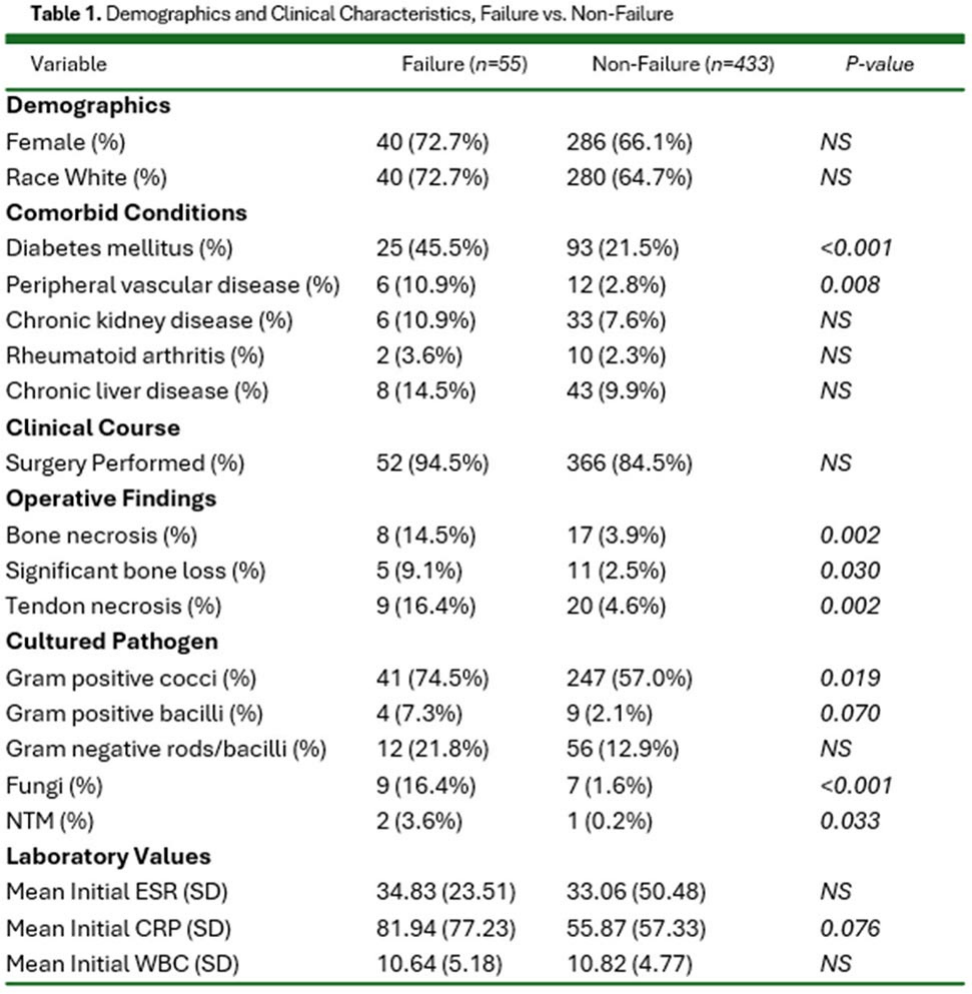

**Methods:**

A retrospective chart review was conducted using inpatient records with ICD10 codes for hand infections generated between 2013-2023 from a major healthcare system in the Southwest United States. 1768 records were found matching these criteria, of which 488 cases were selected at random. Demographic and clinical data was then manually extracted from these charts.

**Results:**

418 patients required surgical intervention during their treatment course (85.65%). No specific comorbidity was associated with higher rates of surgical intervention. Fifty-five patients (11.27%) had recorded treatment failure, defined as readmission of patient after discharge from initial hospitalization, amputation or death. Diabetes and peripheral vascular disease were associated with a higher risk of treatment failure (OR = 3.05; 95% CI: 1.70–5.34; p < 0.001, OR = 4.30; 95% CI: 1.44–11.60; p = 0.008, respectively). Intraoperative findings of bone or tendon necrosis were also associated with a higher risk of treatment failure ((OR = 4.16; 95% CI: 1.62–9.91; p = 0.002, and OR = 4.04; 95% CI: 1.66–9.17; p = 0.002, respectively). 324 patients had a pathogen identified on cultures; gram positive cocci were found in 288 of these patients (88.9%). 105 and 109 patients had MRSA and MSSA identified on growth cultures, respectively. Of our 488 patients, only 293 had a recorded CRP value and 236 had a recorded ESR value. These values were only trended in eight and three patients, respectively (< 2%).

**Conclusion:**

Patients with diabetes and peripheral vascular disease who develop orthopedic hand infections are at a higher risk for treatment failure. Inflammatory markers were not found to be routinely obtained throughout patient treatment course or after completion of treatment.

**Disclosures:**

All Authors: No reported disclosures

